# Auditory-motor adaptation to frequency-altered auditory feedback occurs when participants ignore feedback

**DOI:** 10.1186/1471-2202-14-25

**Published:** 2013-03-09

**Authors:** Dwayne Keough, Colin Hawco, Jeffery A Jones

**Affiliations:** 1 Psychology Department & Laurier Centre for Cognitive Neuroscience, Wilfrid Laurier University, Waterloo, ON, N2L 3C5, Canada

**Keywords:** Internal model, Sensorimotor, Frequency-altered feedback, Auditory feedback, Fundamental frequency, Pitch, Musical training, Singing, Voice, Speech production

## Abstract

**Background:**

Auditory feedback is important for accurate control of voice fundamental frequency (*F*_0_). The purpose of this study was to address whether task instructions could influence the compensatory responding and sensorimotor adaptation that has been previously found when participants are presented with a series of frequency-altered feedback (FAF) trials. Trained singers and musically untrained participants (nonsingers) were informed that their auditory feedback would be manipulated in pitch while they sang the target vowel [/ɑ /]. Participants were instructed to either ‘compensate’ for, or ‘ignore’ the changes in auditory feedback. Whole utterance auditory feedback manipulations were either gradually presented (‘ramp’) in -2 cent increments down to -100 cents (1 semitone) or were suddenly (’constant‘) shifted down by 1 semitone.

**Results:**

Results indicated that singers and nonsingers could not suppress their compensatory responses to FAF, nor could they reduce the sensorimotor adaptation observed during both the ramp and constant FAF trials.

**Conclusions:**

Compared to previous research, these data suggest that musical training is effective in suppressing compensatory responses only when FAF occurs after vocal onset (500-2500 ms). Moreover, our data suggest that compensation and adaptation are automatic and are influenced little by conscious control.

## Background

The goal of speech and singing is the production of sound. The fundamental frequency (*F*_0_), or vocal pitch, is one of the most salient features of the sound produced. For instance, altering the pitch of a vocal production often affects the meaning of the utterance [1]. Several studies have shown that auditory feedback is involved in the control of voice *F*_0._ For example, modifying auditory feedback generally results in compensatory responses in participants’ ongoing vocal productions [2-9].

 There is little doubt that sensory feedback is essential for the acquisition and maintenance of precise motor control [2-7,9-15]. However, recent evidence [16-18] questions the degree of influence of various forms of sensory feedback, specifically auditory feedback, on ongoing motor productions when participants possess extensive training with the task. For instance, Finney and Palmer [16] demonstrated that presenting or removing auditory feedback did not differentially affect the quality of a pianist’s performance when asked to execute a well-rehearsed piece from memory. Other work showed that masking auditory feedback with noise led to a greater number of errors while singing pitch targets in nonsingers than in trained singers [19]. Thus, musical training may contribute to musicians’ precise performance, especially when auditory feedback is altered, masked, or eliminated altogether. One possible explanation is that the differences observed in the control of voice *F*_0_ resulted from an increased reliance on an ‘internal model’ for feedforward control.

 The sensory feedback associated with particular motor movements is thought to be represented by an ‘internal model’. Internal models are proposed to exist as neural maps of skilled movements that store the relationships between the motor commands, environment and sensory feedback for their production [14,20,21]. Mounting evidence regarding the control of limb dynamics [22] and the control of speech [5,7,23] and singing [24-26] suggests that internal models regulate voluntary motor movements. In a previous study, we hypothesized that an internal model for F0 control would be more refined and entrenched in singers than in untrained participants (nonsingers) [24]. In that study, trained singers and nonsingers reproduced a musical target while receiving unaltered or frequency-altered feedback (FAF) that was shifted down by one semitone, without any specific instructions to ignore or compensate to FAF. When individuals are presented with modified sensory feedback regarding any voluntary movement, they typically respond (compensate) by altering their movements in the opposite direction of the manipulation [11,13,14]. Aftereffects, on the other hand, occur when feedback is returned to normal (unaltered), following a series of altered feedback trials. In this case, participants respond as if they ‘anticipate’ the altered feedback, so they briefly make movements that err in the same direction as their compensatory responses [10,15]. In this previous vocal experiment [24], even though all participants compensated for the FAF, aftereffects were only observed in the singers’ data. That is, singers’ *F*_0_ values were higher than their baseline *F*_0_ values when they heard their *F*_0_ returned to normal (i.e., shifted in the opposite direction of the previously experienced FAF), whereas nonsingers *F*_0_ values during testing were similar to those obtained during baseline. Singers’ data also indicated that aftereffects generalized to notes other than the one they sang during training (FAF trials).

 Several studies have suggested that compensation responses are automatic (i.e., reflex-like). Burnett, McCurdy, and Bright [27] found that *F*_0_ response latencies were reduced when participants had immediate control over the onset of FAF that occurred for 100 ms following a button press. However, no differences were observed to the direction, magnitude, or the peak time of voice *F*_0_ responses. By contrast, when Munhall and colleagues [9] presented participants with formant frequency manipulations that coincided with vocal onset, a robust compensation was observed in all conditions even when the researchers instructed speakers to ignore changes in auditory feedback. Moreover, although a number of studies conducted by Larson and colleagues ([2,3,28], but see [29]) did not directly investigate whether participants could ignore FAF, their results suggest that participants compensate for pitch shift manipulations even when told to keep their voice stable and to ignore any auditory feedback variation presented over the headphones. These data suggest that compensation to formant perturbations [9] and FAF [2,3,28,30] is automatic and that compensatory responses do not appear to be modified by a conscious strategy in vocally untrained participants.

 Zarate and Zatorre [17] used functional magnetic resonance imaging (fMRI) to assess the neural processes associated with singing under different feedback conditions. When participants were presented with normal auditory feedback and were instructed to sing various target notes as accurately as possible, the behavioural results indicated that singers were more accurate and less variable in producing the musical targets than nonsingers. Despite the differences in vocal production during the normal feedback condition, both singers and nonsingers exhibited similar functional networks for singing. Zarate and Zatorre [17] also exposed their participants to FAF (2 semitones up or down) in the middle of their utterances (between 1000-1500 ms following vocal onset). When participants were instructed to actively compensate for the FAF, they observed no difference in the level of compensation in singers versus non-musicians. When participants were exposed to FAF and instructed to ignore their feedback (i.e., do not compensate for the manipulations), the pattern of behavioural results indicated that singers suppressed their compensatory responses to the FAF: Singers’ *F*_0_ values during the ignore condition were similar to those obtained when they received unaltered auditory feedback. Non-musicians were unable to ignore the FAF and compensated by altering their *F*_0_ in the opposite direction of the perturbations. Differences were also found in the neural networks activated by singers compared to nonsingers in the ignore condition. In a follow up study, Zarate, Wood and Zatorre [18] found that both singers and non-musicians had difficulty ignoring small perturbations to their auditory feedback (1/4 semitone up or down).

 The results of Zarate and colleagues [17,18] raise an interesting question about the influence of musical training and the use of auditory feedback during voice *F*_0_ control; is it possible that vocally trained participants’ compensatory responses are under conscious control? According to Zarate and Zatorre’s [17] results, it appears that as long as the participants possessed sufficient vocal training, then they were able to consciously suppress compensatory responses to noticeable pitch shifts in their auditory feedback when instructed to do so. It may be that singers were able to weight alternative sources of sensory feedback for vocal control more heavily (e.g., proprioception) during these trials.

 The current study was designed to examine singers’ and nonsingers’ *F*_0_ responses to gradually changing (-2 cent increments to -100 cents, 1 semitone) or constant (-100 cents) FAF at the onset of an utterance. We were particularly interested in whether participants could suppress compensatory responses to FAF manipulations when instructed to do so. That is, we examined participants’ vocal responses when instructed to compensate or maintain (ignore FAF) a steady voice *F*_0_ during FAF trials to determine if compensatory responses could be influenced by volitional control.

 Singers and nonsingers produced target notes at specific frequencies while receiving subtle and large modifications to their auditory feedback. Participants were informed that their auditory feedback would be manipulated in pitch and they were instructed to either (1) ‘compensate’ for these changes by altering their voice *F*_0_ in the opposite direction of the perturbation or (2) to ‘ignore’ their auditory feedback and maintain their voice *F*_0_ at frequencies similar to when their feedback was unaltered. The purpose was to investigate (i) whether task instructions influence the compensatory response and sensorimotor adaptation that are typically observed during FAF studies. Moreover, (ii) collecting data from singers and nonsingers helped identify whether musical training influenced acoustic-motor control when participants were instructed to compensate or ignore auditory feedback manipulations. A third consideration (iii) was to determine if the nature of the pitch-shift stimuli would affect the participants’ ability to ignore FAF changes. To this end, two FAF conditions were included, subtle ramped changes (-2 cents per trial, up to -100 cents) or constant (but abrupt) changes (a -100 shift in FAF). Previous work has suggested participants are not generally aware of small FAF changes such as in the ramped approach [31], while abrupt -100 cent shifts should be immediately noticed by all participants. Regardless of whether the pitch manipulations were ramped or abrupt and then constant, if both singers and nonsingers were able to suppress or eliminate compensatory responses and sensorimotor adaptation, this would suggest that these responses are, to a certain degree, subject to volitional control. Conversely, if similar patterns of compensatory responses and sensorimotor adaptation were observed in both singers and nonsingers, this would suggest that these responses are automatic.

 Based on our previous work [24-26], it was hypothesized that both singers and nonsingers would be unable to ignore subtle shifts (-2 cent increments to -100 cents; ‘ramp condition’) in their auditory feedback. As a result, it was expected that participants would exhibit similar patterns of compensatory responding and sensorimotor adaptation during the ramp condition. When auditory feedback was suddenly shifted to -100 cents (‘constant condition’), it was hypothesized that nonsingers would immediately compensate by increasing their voice *F*_0_ in the opposite direction of the manipulation. Over time, it was believed that nonsingers would exhibit sensorimotor adaptation while compensating for the FAF. However, based on the results of Zarate and Zatorre [17], we hypothesized that trained singers would be able to ignore the abrupt changes in auditory feedback when instructed to do so, which would also suggest they are able to maintain existing internal models in the presence of feedback alterations. However, one important distinction between Zarate and Zatorre’s study [17] and the present study is that we presented pitch shifts at utterance onset, as opposed to mid-utterance. Previous work from our group [32] has suggested that different mechanisms may exist for responding to feedback changes mid-utterance versus at utterance onset. It is therefore possible that singers would be unable to ignore FAF changes in our study due to the presentation of FAF at utterance onset.

## Methods

### Participants

Thirty Wilfrid Laurier University students (all women) whose first language was North American English participated in this FAF study. Men were not included so that all participants could adequately sing the same target notes. Of the 30 participants, 15 were trained singers recruited from the Faculty of Music (vocal majors) at Wilfrid Laurier University (mean music training was approximately 12 years). None of the trained singers reported having ‘perfect’ pitch. The remaining 15 participants were nonsingers, such that none had any form of previous vocal training or were currently participating in formal singing. All participants received financial compensation for their time and informed consent was collected from each participant. The Wilfrid Laurier University Research Ethics Committee approved the procedures.

### Apparatus

#### Participant recording sessions

Participants were situated in a double-walled sound attenuated booth (Industrial Acoustic Company, Model 1601-01) and were fitted with headphones (Sennheiser HD 280 Pro) and a condenser microphone (Countryman Isomax E6 Omnidirectional Microphone), which was positioned approximately 3 cm from their mouth. Multitalker babble noise (Auditec, St. Louis, MO) was presented at 70 dB SPL (sound pressure level) to limit natural acoustic feedback. Note that the multitalker babble was unintelligible to the listener, as it consisted of 20 young adults simultaneously reading different passages. The target notes consisted of a female voice singing the vowel-consonant [/ɑ/], that was presented at 220.00 (A3), 246.94 (B3), 293.66 (D4) or 329.63 (E4) Hz, respectively. Microphone signals were sent to a signal processor (VoiceOne 2.0, TC Helicon) that manipulated auditory feedback. The signal processing introduced an unnoticeable delay of 10 ms. The altered feedback was then mixed (Mackie ONYX 1640) with the multi-talker babble and subsequently sent to the participant. Vocal productions were digitally recorded (TASCAM HD-P2) at a sampling rate of 44.1 kHz for future analysis.

#### Target stimuli recording

A trained singer produced the respective targets, A3, B3, D4, and E4, which were processed using the speech modification algorithm STRAIGHT [Speech Transformation and Representation using the Adaptive Interpolation of weighted spectrum; [33] to ensure that each target was exactly 220.00, 246.94, 293.66, or 329.63 Hz.

### Procedure

**Figure 1:**
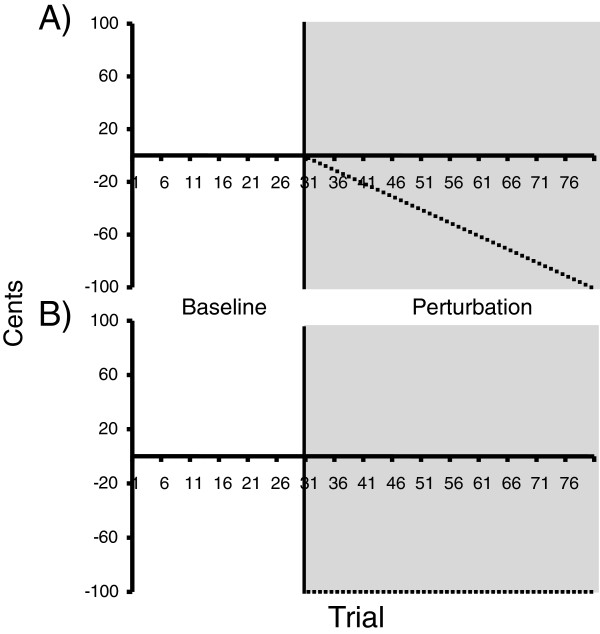
**Depiction of the gradual and constant frequency-altered feedback (FAF) conditions.** During baseline (trials 1-30), the pitch of auditory feedback was not manipulated. During perturbation (FAF) trials, presented in gray, auditory feedback was manipulated from trial 31 (solid vertical line) to trial 80 (all FAF trials represented by the dashed line). (**A**) Auditory feedback was gradually manipulated downwards, in -2 cent increments across trials until auditory feedback was shifted by -100 cents (1 semitone). (**B**) Auditory feedback was shifted by a constant value (-100 cents) for all FAF trials.

Participants were asked to match the pitch of their voice to a musical target (e.g., either A3, B3, D4, or E4) during four conditions that consisted of 80 trials (1 block) per musical note. The order of the target notes was the same for each participant, however the instructions (e.g., ignore or compensate) and manipulations (e.g., ramp or constant shifts) were counterbalanced across subjects. Each block consisted of 30 baseline and 50 FAF trials (see Figure 1 for an outline of the methods). For instance, on the first block of 80 trials participants reproduced the musical target A3 (220 Hz). Participants received natural acoustic (unaltered) feedback during the 30 baseline trials, followed by 50 FAF trials. During the FAF trials, auditory feedback was either gradually shifted downward (ramp condition) in -2 cent increments per trial down to -100 cents, or it was abruptly shifted down (constant condition) 100 cents for all 50 FAF trials. Note that auditory feedback was shifted from the beginning of each utterance until the end of the vocal productions during the FAF trials.

 Participants were instructed to either ‘compensate’ or ‘ignore’ any changes in auditory feedback that may occur during the study. During the ‘compensate’ condition, participants were informed that they would be presented with a musical target at the beginning of each trial. The goal for participants was to match the pitch of their voice as accurately as possible to the target presented. Participants were informed that they would initially receive normal, unaltered, auditory feedback. That is, what they produced would be exactly what they would hear in the headphones. Additionally, they were told that at some point the pitch of their voice presented via the headphones would be different than what they actually produced. Thus, they were instructed to continually monitor their auditory feedback and to try to match what they heard in the headphones to the target presented at the beginning of each trial. Essentially, participants were instructed to compensate for the FAF.

 During the ‘ignore’ condition, participants were informed that they would be presented with a musical target at the beginning of each trial. The goal for participants was to match the pitch of their voice to the target presented, but to ignore their auditory feedback. They were told that initially they would receive normal, unaltered, auditory feedback. However, they were informed that at some point the pitch of their voice presented via the headphones would be different than what they actually produced. Participants were instructed to ignore the FAF (their auditory feedback) and to produce the target consistently, at the same pitch as when their feedback was unaltered. Thus we asked participants to ‘not compensate’ for the pitch shift manipulations, so sounding ‘off’ would be acceptable during these trials. At no time during the study were the participants informed of the direction of the FAF manipulation, at what trial the perturbation occurred, or made aware of alternative strategies that could be used to assist with vocal control.

 An individual trial commenced with the presentation of the target stimulus, which lasted for 1000 ms. Immediately following the termination of the target, multitalker babble was presented for 3500 ms. During the presentation of the multitalker babble, participants were instructed to reproduce the target as accurately (with respect to their instructions) as possible in pitch and duration. A 1000 Hz beep coincided with the last 500 ms of the multitalker babble, which served to signify that the trial was about to conclude and participants should cease vocalization. There was an intertrial interval (ITI) of 3000 ms between trials. Trials were initiated and controlled by a computer and *F*_0_ values for each vocal production were calculated, during offline analyses, using an autocorrelation algorithm included in the Praat program [34]. *F*_0_ values were normalized to each target note (A3, B3, D4 or E4) by calculating the appropriate cent values using the following formula:

$$\mathrm{Cents}=100\left(12\;\log2\;F/B\right)$$

Where F is the *F*_0_ value in Hertz and B is frequency of the target pitch participants heard (220.00, 246.94, 293.66, or 329.63 Hz).

### Statistical analyses

Two measures were calculated to determine the effects of our experimental manipulation on vocal production: a measure of ‘compensation’, and a measure of ‘adaptation’. As it has been previously shown that compensatory responses to FAF typically occur between 130 to 500 ms post perturbation onset [3], the median *F*_0_ value of the first 1500 ms of each vocal utterance was calculated to index the ‘compensation’ response. For adaptation responses, previous studies examined aftereffects following a series of FAF trials [6,7], e.g., [24,35]. Evidence from our laboratory [25,32] demonstrated that sensorimotor adaptation can be observed within 50 ms of vocal onset during exposure to FAF. For instance, although in a recent study we failed to find aftereffects when we examined vocal productions following FAF trials [25], we were able to show that participants initiated vocal productions at frequencies closer to the intended target (i.e., in the direction opposite of the FAF). As a result, the median *F*_0_ value within 50 ms of vocal onset was calculated for each utterance as a measure of vocal-motor adaptation. It should be noted that the adaptation and compensation response might not be completely independent measures, as changing initial *F*_0_ would have an effect on later portions of the utterance.

 To analyze compensation for the FAF, each experimental session was divided into 16 blocks of five trials: 6 blocks of baseline trials (1-5, 6-10, 11-15, 16-20, 21-25, 26-30), and 10 blocks of FAF trials (1-5, 6-10, 11-15, 16-20, 21-25, 26-30, 31-35, 36-40, 41-45, 46-50). The mean of the median *F*_0_ values for both 1500 ms (compensation) and 50 ms (adaptation) was calculated for each block. Dividing trials into blocks was done to reduce inter-trial variability in the data due to inaccuracies in vocal production, and to facilitate visualization of the progression of *F*_0_ changes during each session. Separately for both the adaptation (50 ms) and compensation (1500 ms) results, a mixed ANOVA with 2 (group: singers and nonsingers) × 2 (instruction: ignore or compensate) × 2 (manipulation: ramp or constant) × 16 (block) factors was conducted. Tukey’s honestly significant difference (HSD) test was used for post-hoc analyses with an alpha level of .05 for all statistical tests.

## Results

### Compensatory responses

**Figure 2:**
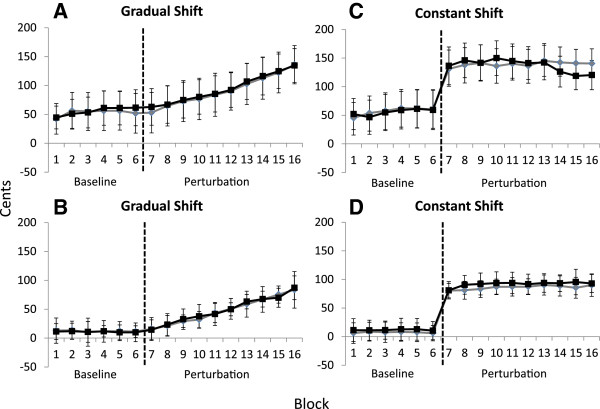
**Average fundamental frequency (*F***_***0***_**) values across blocks (5 trials per block) of unaltered (baseline) and frequency-altered feedback (FAF) trials when participants were instructed to ‘compensate’ or ‘ignore’ changes in auditory feedback.***F*_*0*_ was calculated relative to the target note from each block. The median *F*_*0*_ value was calculated for the initial 1500 ms of vocal productions. The vertical dotted line indicates the commencement of FAF trials. (**A**) Nonsingers’ median 1500 ms data across blocks of unaltered and FAF trials, when auditory feedback was gradually (-2 cent increments across trials to -100 cents) shifted in pitch. Black squares represent nonsingers’ *F*_0_ values when instructed to ‘compensate’ for FAF. Gray diamonds represent nonsingers’ *F*_0_ values when instructed to ‘ignore’ the FAF. Error bars depict standard error of the mean. (**B**) Singers’ median 1500 ms data across blocks of unaltered and FAF trials, when auditory feedback was gradually (-2 cent increments across trials to -100 cents) shifted in pitch. Black squares represent singers’ *F*_0_ values when instructed to ‘compensate’ for FAF. Gray diamonds represent singers’ *F*_0_ values when instructed to ‘ignore’ the FAF. Error bars depict standard error of the mean. (**C**) Nonsingers’ median 1500 ms data across blocks of unaltered and FAF trials, when auditory feedback was constantly shifted down by -100 cents. Black squares represent nonsingers’ *F*_0_ values when instructed to ‘compensate’ for FAF. Gray diamonds represent nonsingers’ *F*_0_ values when instructed to ‘ignore’ the FAF. Error bars depict standard error of the mean. (**D**) Singers’ median 1500 ms data across blocks of unaltered and FAF trials, when auditory feedback was constantly shifted down by -100 cents. Black squares represent singers’ *F*_0_ values when instructed to ‘compensate’ for FAF. Gray diamonds represent singers’ *F*_0_ values when instructed to ‘ignore’ the FAF. Error bars depict standard error of the mean.

The median 1500 ms *F*_0_ values for singers and nonsingers are displayed in Figure 2. The ANOVA conducted on the median 1500 ms *F*_0_ values with 2 (group: singers and nonsingers) × 2 (instruction: ignore or compensate) × 2 (manipulation: ramp or constant) × 16 (block) factors revealed a marginal effect of group, *F*(1, 28) = 3.17, *p* = .086. On average, singers’ median 1500 ms *F*_0_ values across baseline and FAF trials were lower, or more consistently near the target frequency, than nonsingers’ median 1500 ms *F*_0_ values. Significant main effects of manipulation, *F*(1, 28) = 28.08, *p* < .05, and block, *F*(15,420) = 110.64, *p* < .05 were also observed. As one would expect, overall, the *F*_0_ values obtained for all participants during the ramp manipulation were significantly lower than the *F*_0_ values obtained during the constant manipulation. In regard to the main effect of block, post-hoc testing indicated that the average *F*_0_ values obtained during the baseline trials for both ramp and constant manipulations were found to be significantly smaller than the average *F*_0_ values obtained during all FAF blocks of trials for both ramp and constant manipulation conditions, (all *p*’s < .05).

A significant two-way interaction between manipulation and block was also found, *F*(15,420) = 75.71, *p* < .05. The post hoc analysis revealed that the average baseline blocks (1-6) of *F*_0_ values obtained during the ramp manipulation condition were significantly different than the average *F*_0_ values obtained during FAF blocks 9-16. As the pitch shift manipulation became progressively larger, participants, on average, compensated by increasing the pitch of their voice in the opposite direction of the manipulation so that their *F*_0_ values were different than those produced during the baseline trials. Also, as the FAF during the ramp condition was progressively shifted lower, participants’ *F*_0_ values gradually became significantly different than those observed on previous blocks of FAF trials (*p* < .05). For instance, the *F*_0_ values obtained on the last block (block 16) of FAF trials during the ramp manipulation condition were significantly higher than all *F*_0_ values obtained on previous blocks of FAF trials (*p* < .05). With respect to the *F*_0_ values obtained during the constant manipulation condition, post hoc testing indicated that the baseline values were significantly lower than the *F*_0_ values obtained during all FAF blocks (7-16) of trials (*p* < .05). Interestingly, the *F*_0_ values observed during the initial block (7) of FAF trials were not statistically different than the *F*_0_ values observed on any other block (8-16) of FAF trials (*p* > .05). Therefore, singers and nonsingers, on average, compensated immediately and consistently to the sudden and constant change (-100 cents) in auditory feedback across all blocks of FAF trials. Although the *F*_0_ values collected on the initial block (7) during the constant manipulation condition were significantly higher than those values collected on FAF blocks 7-14 of the ramp manipulation condition (all *p*’s < .05), they were not significantly different than the *F*_0_ values obtained on blocks 15 and 16 of the ramp condition. The level of compensation to FAF was similar when auditory feedback was manipulated downward between 80-100 cents across both conditions. Lastly, no significant effect of instruction (ignore or compensate conditions) was observed in the median 1500 ms *F*_0_ values, *F*(1, 28) = .011, *p* > .05. Even when instructed to ignore the FAF, participants were unable to maintain their voice *F*_0_ at similar levels to those obtained during baseline ramp and constant conditions. No other significant main effects or interactions were observed.

### Adaptation responses: differences between singers and nonsingers

**Figure 3:**
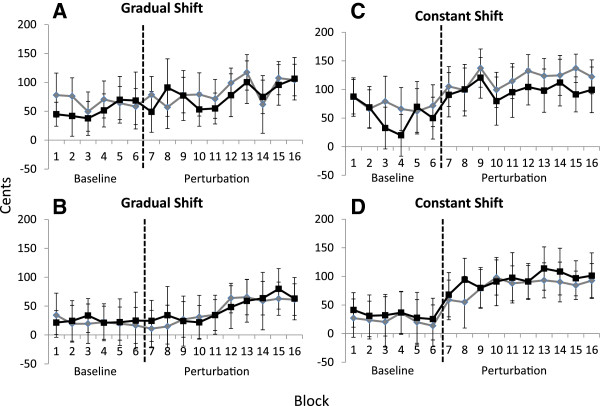
**Average fundamental frequency (*F***_***0***_**) values across blocks (5 trials per block) of unaltered (baseline) and frequency-altered feedback (FAF) trials when participants were instructed to ‘compensate’ or ‘ignore’ changes in auditory feedback.** The median *F*_*0*_ value was calculated for the initial 50 ms of vocal productions *F*_*0*_, normalized to the target note from each block. The vertical dotted line indicates the commencement of FAF trials. (**A**) Nonsingers’ median 50 ms data across blocks of unaltered and FAF trials, when auditory feedback was gradually (-2 cent increments across trials to -100 cents) shifted in pitch. Black squares represent nonsingers’ *F*_0_ values when instructed to ‘compensate’ for FAF. Gray diamonds represent nonsingers’ *F*_0_ values when instructed to ‘ignore’ the FAF. Error bars depict standard error of the mean. (**B**) Singers’ median 50 ms data across blocks of unaltered and FAF trials, when auditory feedback was gradually (-2 cent increments across trials to -100 cents) shifted in pitch. Black squares represent singers’ *F*_0_ values when instructed to ‘compensate’ for FAF. Gray diamonds represent singers’ *F*_0_ values when instructed to ‘ignore’ the FAF. Error bars depict standard error of the mean. (**C**) Nonsingers’ median 50 ms data across blocks of unaltered and FAF trials, when auditory feedback was constantly shifted down by -100 cents. Black squares represent nonsingers’ *F*_0_ values when instructed to ‘compensate’ for FAF. Gray diamonds represent nonsingers’ *F*_0_ values when instructed to ‘ignore’ the FAF. Error bars depict standard error of the mean. (**D**) Singers’ median 50 ms data across blocks of unaltered and FAF trials, when auditory feedback was constantly shifted down by -100 cents. Black squares represent singers’ *F*_0_ values when instructed to ‘compensate’ for FAF. Gray diamonds represent singers’ *F*_0_ values when instructed to ‘ignore’ the FAF. Error bars depict standard error of the mean.

The median 50 ms *F*_0_ values for singers and nonsingers were calculated for each condition and are displayed in Figure 3. The ANOVA conducted on the median 50 ms * F*_0_ values with 2 (group: singers and nonsingers) × 2 (instruction: ignore or compensate) × 2 (manipulation: ramp or constant) × 16 (block) factors revealed a significant main effect of manipulation, *F*(1, 28) = 8.80, *p* < .05, and block, *F*(15, 420) = 15.36, *p* < .05. On average, participants’ median 50 ms *F*_0_ values during the ramp manipulation condition were significantly lower than the 50 ms *F*_0_ values during the constant manipulation condition. Post hoc results on the main effect of block revealed that participants’ *F*_0_ values during baseline trials were not significantly different (*p* > .05). On the other hand, the *F*_0_ values obtained during baseline (blocks 1-6) were found to be significantly lower than the *F*_0_ values obtained during FAF blocks 12-16 (all *p*’s < .05). Moreover, some of the baseline *F*_0_ values (blocks 3, 4 & 6) were significantly lower than the *F*_0_ values observed on FAF blocks 8-16 (all *p*’s < .05). Thus, sensorimotor adaptation was observed when participants were subjected to FAF, regardless of whether they were instructed to compensate or ignore the manipulated auditory feedback.

 A significant two-way interaction was also observed between manipulation and block, *F*(15, 420) = 3.62, *p* < .05. Participants’ baseline *F*_0_ values during the ramp manipulation condition were not statistically different nor were they different than the baseline *F*_0_ values obtained during the constant manipulation condition (all *p*’s > .05). However, the *F*_0_ values observed during the baseline trials in the ramp condition were significantly lower than those values observed during FAF blocks 13, 15 and 16 (*p* < .05). As participants’ auditory feedback was gradually shifted downward, they progressively increased their voice *F*_0_ so that they initiated vocal productions at levels closer to the intended target frequency. Similar results were also observed in the median 50 ms *F*_0_ values during the constant manipulation condition. Baseline *F*_0_ values for blocks 2-6 were found to be significantly lower than the *F*_0_ values on FAF blocks 8-16 (all *p*’s < .05). Lastly, the main effect of instruction was not significant, *F*(1, 28) = .03, *p* > .05. Participants’ voice *F*_0_ values were not statistically different across all blocks of trials, regardless of whether they were instructed to compensate or ignore the FAF. No other significant main effects or interactions were observed.

## Discussion

 The purpose of the present study was to determine whether instructing participants to ‘compensate’ or ignore’ gradual (-2 cent increments per trial down to -100 cents) or constant changes (-100 cents) in auditory feedback could result in the voluntary suppression of compensatory responses and sensorimotor adaptation. Regardless of whether participants received the gradual or constant pitch manipulation, both singers and nonsingers could not intentionally suppress the compensatory response during FAF trials. The pattern of compensation observed when participants were instructed to ‘ignore’ the FAF was indistinguishable from the compensatory responses observed when they were instructed to ‘compensate’ for the FAF. Additionally, participants’ median 50 ms *F*_0_ values suggested that the level of sensorimotor adaptation that occurred during the ‘ignore’ condition was similar to the adaptation observed during the ‘compensate’ condition. Voice *F*_0_ values observed throughout the FAF (gradual and constant) trials indicates that both singers and nonsingers updated their internal models by adjusting their *F*_0_ so that they initiated their vocal productions at frequencies closer to the intended target.

 When participants were instructed to ‘compensate’ for the gradual presentation of FAF they correspondingly adjusted their *F*_0_ in the opposite direction of the manipulation. That is, within 1500 ms of vocal onset, both singers and nonsingers increased their voice *F*_0_ in the opposite direction of the perturbations in order to maintain pitch accuracy with the intended target (see Figure 2). Participants also exhibited sensorimotor adaptation while compensating for the gradual FAF manipulation. Participants’ data within 50 ms of their vocalization onset indicates that as the pitch manipulation progressively decreased, they recalibrated their internal models to initiate vocal productions at increasingly higher frequencies. In other words, participants adjusted their entire vocal production and initiated subsequent utterances at *F*_0_ values similar to those produced on previous FAF trials. In doing so they maintained consistency in their vocal-motor plan for how they sang musical notes. This suggests that as participants were gradually compensating for the FAF, by changing their *F*_0_ values in the opposite direction of the perturbation, they were also continually updating their internal models to account for the gradual decrease in the F0 of their auditory feedback.

 Similarly, when participants were instructed to compensate for the sudden and large change (constant condition) in their auditory feedback, results indicated that participants increased their *F*_0_ in the opposite direction of the manipulation. Interestingly, the *F*_0_ values, on average, obtained during the first block of FAF trials were not statistically different than the *F*_0_ values obtained on any other block of FAF trials. Thus, participants compensated to a similar degree across all FAF trials when presented with a constant shift (1 semitone) in auditory feedback. Furthermore, participants’ median 50 ms *F*_0_ data indicated that sensorimotor adaptation occurred. As singers and nonsingers rapidly compensated for the FAF, they also adjusted their internal models to initiate *F*_0_ values closer to the intended target. *F*_0_ values were determined to be significantly different than the baseline F0 values from the second block (trials 6-10) of FAF trials onward. As a consequence, instructing participants to compensate for FAF resulted in similar responses to those observed previously in our laboratory [25] and by others using the FAF paradigm [2,3,6,7,17,18,35-37].

The finding that compensatory responses are not easily suppressed by instructions to ignore feedback is consistent with previous studies using FAF [29], formant frequency manipulations [9], and masking noise [38]. A recent study by Munhall and colleagues [9] found that participants rapidly compensated for formant frequency manipulations when they were instructed to ignore the modified feedback. Moreover, when the manipulations were removed participants exhibited aftereffects. As a consequence, Munhall and colleagues [9] suggest that their data do not necessarily provide “evidence of a fixed-response system that cannot be adjusted with practice or strategies”, but rather argue that compensatory responses to vowel modifications are not strategic responses to the detection of auditory feedback manipulations. This is also congruent with the findings from the current study; however, it is uncertain whether repeated exposure (‘practice’) to subtle (2 cents) manipulations in auditory feedback would result in the voluntary suppression of compensatory responses to FAF.

 For instance, when similar pitch shift values were presented incrementally across trials in previous studies from our laboratory (+/-2 cents and +/- 4 cents) e.g., [25], participants stated that they were unaware that their voice was manipulated in pitch. Munhall and colleagues also indicated that participants possessed no particular knowledge of the nature of the manipulation when formants were modified in small increments trial-by-trial e.g., [39]. Indeed, it has been reported that an early automatic response to unexpected changes in auditory feedback occurs [2,3,29]. If this response assists with small, unexpected perturbations (as opposed to larger more obvious changes in auditory feedback) then the presentation of gradual shifts in auditory feedback may fall within a certain automatic compensatory range that cannot be suppressed voluntarily, nor may it require the ‘conscious’ detection of the error for the compensatory response to occur; see also [18]. This is consistent with the results of Loui et al. [40], who reported that amusic (‘tone-deaf’) participants were able to reproduce the pitch direction of two successive single tones, although they were at chance discriminating pitch direction. That is, although amusics have difficulty perceptually identifying pitch changes that are smaller than a semitone [41], they are capable of producing the correct pitch direction as accurately as controls [40]. This supports Loui et al.’s [40] notion that the auditory pathway responsible for vocal production may be distinct from the pathway responsible for conscious perception. Thus, compensating for altered feedback may occur without a participant’s awareness. Alternatively, it is possible that repeated exposure to large changes (e.g., 100, 200 cents) in auditory feedback may allow compensatory responses to be voluntarily controlled [17], e.g., [29]. Regardless, the data presented by Munhall et al. [9] and the results of the current study suggest that motor preparation, initiation, and production of vocal utterances are heavily influenced by auditory feedback. Moreover, instructing participants to ignore changes in feedback does not appear to influence compensatory responding or alter the pattern of sensorimotor adaptation.

Auditory feedback has been shown to be important for accurate *F*_0_ control, and it has also been shown to be influential during the acquisition of a novel musical piece. Finney and Palmer [16] found that trained pianists performance improved when auditory feedback was provided while learning a novel song. However, when the musicians were required to produce a well-rehearsed piece from memory, the removal of auditory feedback had no effect on performance [16]. Similar to the trained singers in Zarate and Zatorre’s study [17], who could suppress compensatory responses to +/-200 cents (2 semitone) manipulations, it appears that musical training may allow musicians to perform in the absence or modification of auditory feedback. In regards to singing, one possibility is that presenting the pitch manipulations so they occur later into vocal production [such as 1000-1500 ms after vocal onset, which Zarate and Zatorre 17 used] may result in easier identification of FAF (e.g., efference copy violation), or it may allow for singers to rely on alternative components (e.g., muscle memory, kinesthetic feedback) of their internal model to suppress compensatory responding.

Conceptually, internal models are hypothesized to compare sensory feedback with motor acts by means of a comparator examining differences between perception and production. These differences are hypothesized to be computed based on a corollary discharge, such that the output of an internal model maps the motor commands (e.g., efference copy) with the expected sensory feedback from the actions. When a match exists between perception and production, the result is a net cancellation of the sensory input, which in turn causes a dampened sensory experience [42]. Conversely, when there is a discrepancy between the perception and production of a motor act, the corollary discharge does not dampen the sensory feedback. As a consequence, there is an intensification of the sensory experience that potentially alerts us to environmental events [42].

For instance, in a series of event related potential (ERP) and magnetoencephalographic (MEG) studies using FAF, Heinks-Maldonado and colleagues [42,43] found that an early sensory detection component (e.g., M100, which occurs approximately 100-150 ms following auditory stimuli) generated in the auditory cortex was maximally suppressed when a participant heard his own unaltered voice. When participants received pitch-shifted feedback the researchers observed an increase in the amplitude of the M100 relative to when they received unaltered auditory feedback [43]. Participants in the current study may have also exhibited similar cortical activity when presented with FAF, as they initiated compensatory responses. However, presenting the pitch manipulations so they coincide with vocal onset may make compensatory responses more difficult to suppress than if the FAF was to be presented mid-utterance.

When FAF is delivered mid-utterance [17], e.g., [29], the efference copy associated with the motor commands is not initially violated, as the participant *initially* hears exactly what they are producing. When the FAF occurs, it is possible that the nervous system has already determined that the motor commands are appropriate for the target note produced and that the error perceived is due to something external (e.g., the experimenter). A study from our group [32] directly addressed this issue. FAF was either presented at utterance onset or mid-utterance, and in some cases the mid-utterance FAF was induced by removing ongoing FAF (which was present from utterance onset). The results of this study showed that the compensation response to FAF at utterance onset was much larger than the response to mid-utterance FAF. Furthermore, the amplitude of compensation to removing FAF mid-utterance was identical to initiating FAF mid-utterance, indicating participants viewed the removal of ongoing FAF in a similar way as the introduction of an FAF change. The results of this study were taken as evidence for different mechanisms for vocal control at utterance onset (where the goal is to achieve a target pitch) and during mid-utterance (where the goal is to maintain pitch at a steady level). The mechanism for vocal control at utterance onset likely involves the efference copy, whereas the mid-utterance mechanism can rely more exclusively on ongoing auditory feedback. This is relevant when comparing the results of this study, which introduced FAF at utterance onset, and the results of Zarate and Zatorre’s study [17], which introduced FAF mid-utterance. Given the extensive experience trained singers possess with vocal control, participants in Zarate and Zatorre’s [17] study may have relied more on kinesthetic feedback (e.g., vocal-fold positioning) to maintain the pitch of their voice during FAF trials, whereas nonsingers, possibly due to their lack of formal music training, were unable to suppress compensatory responses. On the other hand, when the FAF coincides with vocal onset, at no point does the perceived sensory feedback match the sensory feedback predicted by the participants' internal model, resulting in an intensified sensory experience (efference copy violation) e.g., [42,43]. Regardless of whether participants were aware of the FAF manipulations, compensatory responses were initiated to subtle and large changes in auditory feedback. This suggests that once the efference copy has been violated, participants’ internal models are automatically adjusted and compensatory responses are initiated in an attempt to offset the deviant auditory feedback.

There is substantial evidence that auditory feedback is influential in achieving precise vocal control. Murbe et al. [44] and Larson et al. [45] have also demonstrated that kinesthesia substantially contributes to singers’ pitch control at the beginning of an utterance (< 100 ms). After 100 ms, auditory feedback participates in *F*_0_ control [45]. However, Munhall and colleagues [9] found that instructing participants to rely on the kinesthetic properties for *F*_0_ control was insufficient to suppress compensatory responding to formant frequency manipulations. As the participants in Munhall et al. [9] were not musically trained then it may be that they were unable to utilize the kinesthetic feedback as efficiently as trained singers to suppress compensatory responses.

 Our results and those of others [17,18,24-26,29] suggest that the processes involved in comparing the actual sensory consequences with the expected sensory consequences during vocalization is dependent on various forms of sensory feedback (e.g., auditory, kinesthetic). Singers’ ability to ignore FAF e.g., [17] may result from relying less on auditory feedback and more on alternative feedback strategies (e.g., use of kinesthetic feedback) once a musical piece has been memorized [16]. Alternatively, a more likely explanation is that participants may utilize the information they receive following vocal onset differently to maintain a stable voice *F*_0_ as opposed to the information they receive mid-utterance [32]. Overall, it appears that vocal training may only be effective in suppressing compensatory responses to FAF in instances where the perturbations are presented mid utterance.

## Conclusion

 Results from the present study suggest that neither musically trained singers nor nonsingers can voluntarily suppress compensatory responses to gradual or large FAF manipulations. Sensorimotor adaptation was also observed during both ignore and compensate conditions. Formal music training appears to be useful in suppressing compensatory responses only when the FAF is presented following vocal onset (e.g., 500-2500 ms) [17,29]. In sum, it appears that compensation and adaptation to FAF are automatic and are influenced little by ‘conscious’ control, providing the FAF manipulation coincides with vocal onset.

## Competing interests

All authors declare they have no competing interests.

## Authors’ contributions

DK, CH and JAJ conceived, and designed the experiments. DK collected the data. DK and JJ analyzed the data and wrote the paper. All authors approved the final draft.
